# Evaluation of insulin sensitivity by hyperinsulinemic-euglycemic clamps using stable isotope-labeled glucose

**DOI:** 10.1038/s41421-018-0016-3

**Published:** 2018-04-17

**Authors:** Yuanyuan Zhang, Lina Xu, Xiaohui Liu, Yiguo Wang

**Affiliations:** 1 MOE Key Laboratory of Bioinformatics, Tsinghua-Peking Center for Life Sciences, School of Life Sciences, Tsinghua University, Beijing, 100084 China; 2 National Protein Science Technology Center, Tsinghua University, Beijing, 100084 China

Dear Editor,

Insulin resistance is a critical factor in the pathogenesis of metabolic diseases such as obesity, nonalcoholic fatty liver disease (NAFLD) and type 2 diabetes (T2D)^1^. For many years, the hyperinsulinemic-euglycemic clamp has been used as a “gold standard” method to accurately measure insulin action in vivo^2^. It is widely used in humans, dogs, rats and mice. During the clamp, glucose kinetics, including the rates of endogenous glucose production and disposal in mice, are conventionally assessed with tracers^3^. The radioactive tracer [3-^3^H]glucose is commonly used because it is sensitive and massless, but it is harmful to our environment, and cannot be used in humans because it is hazardous if introduced into the body^3^. Therefore, medical research has turned to stable isotopes as alternative tracers. Although many studies have successfully established the clamp method using stable isotopes in humans, no method has been developed for laboratory mice because of the limitations of mass spectrometry, which requires the infusion of a large dose of stable isotope and a large volume of blood^4–8^.

In this study, we have successfully devised a sensitive method using [6,6-^2^H]glucose as a tracer in mice. [6,6-^2^H]glucose is a stable (non-radioactive) naturally occurring isotope with no known harmful effects and similar metabolic effects to normal glucose^5^. It can be distinguished from natural isotopomers of glucose (i.e., with other isotopic fine structures) using a high resolution mass spectrometer. To establish this method, we tested it in a high-fat diet (HFD)-induced obese mouse model, which is well known and widely used in metabolism research^9–12^. Compared to mice fed with a regular diet (RD), HFD-induced obese mice had significantly higher body weight, plasma insulin levels, glucose production measured by pyruvate tolerance test (PTT), glucose intolerance evaluated by glucose tolerance test (GTT) and insulin insensitivity assessed by insulin tolerance test (ITT) (Fig. 1a–e). All the results indicate that glucose production and insulin resistance were dramatically increased in HFD-fed mice compared to RD-fed animals.

**Fig. 1:**
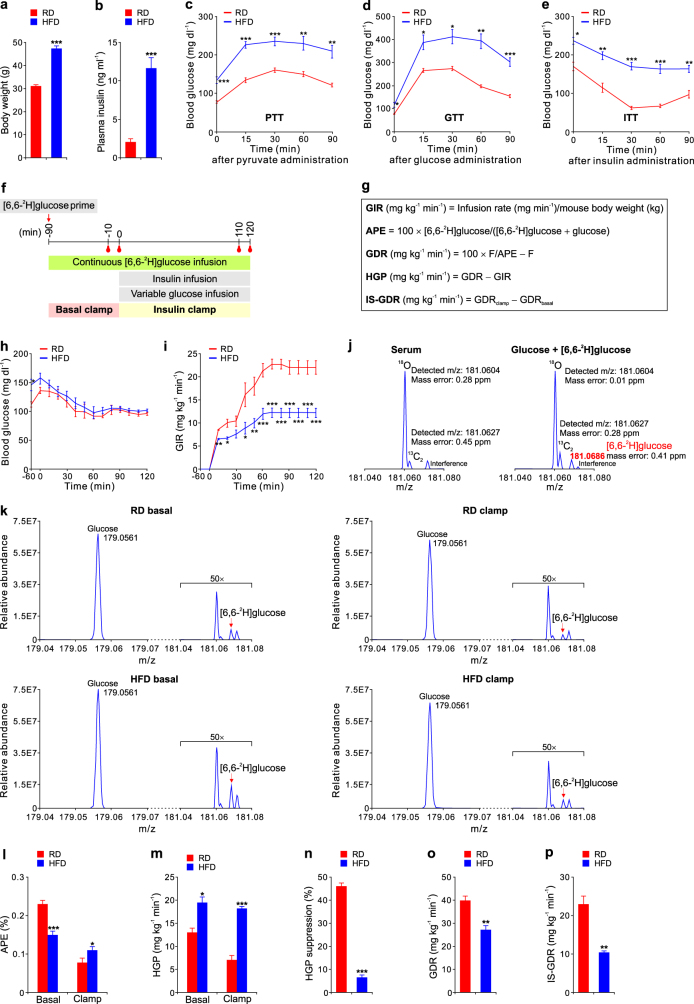
Evaluation of insulin sensitivity by hyperinsulinemic-euglycemic clamps using [6,6-^2^H]glucose in mice. **a**–**e** Comparison of body weight (**a**), plasma insulin levels (**b**), pyruvate tolerance test (PTT, **c**), glucose tolerance test (GTT, **d**) and insulin tolerance test (ITT, **e**) in mice fed with a regular diet (RD) and a high-fat diet (HFD) for 16 weeks. **f** A time-line of the procedure for performing the insulin clamp. Blood samples were taken at time −10, 0, 110, and 120 min for further analyses. **g** The equations used to calculate and evaluate insulin sensitivity. **f** indicates the constant isotope infusion rate. **h, i** Blood glucose levels (**h**) and glucose infusion rate (GIR, **i**) measured during clamp studies. **j** Representative MS spectra showing the isotopic fine structures of glucose (M+2) in mouse serum and in a standard mixture of glucose (1 mg ml^−1^) and [6,6-^2^H]glucose (1 μg ml^−1^). **k** Representative MS spectra of monoisotopic peaks and M+2 isotopic fine structures of glucose with infused [6,6-^2^H]glucose extracted from the serum of mice fed a RD or HFD for 16 weeks. The intensity of the M+2 isotopic envelopes is magnified by 50-fold on each spectrum. All peaks are displayed using relative abundance on the same scale. **l**, **m** Atom percent excess (APE, **l**) and hepatic glucose production (HGP, **m**) under basal and clamp conditions are shown. **n**–**p** HGP suppression (**n**), glucose disposal rate (GDR, **o**), and insulin-stimulated GDR (IS-GDR, **p**) are shown. Data are shown as mean ± s.e.m. *P < 0.05, **P < 0.01, ***P < 0.001, *n* = 5

To evaluate insulin action and glucose metabolism in vivo, we performed hyperinsulinemic-euglycemic clamp studies using [6,6-^2^H]glucose as a tracer (Fig. 1f, g). A bolus of [6,6-^2^H]glucose (600 μg kg^−1^) was administered via catheter followed by continuous infusion of [6,6-^2^H]glucose at the rate of 30 μg kg^−1^ min^−1^ for 90 min to maintain steady-state conditions. During the clamp, we monitored the blood glucose levels, plasma insulin levels and glucose infusion rate (GIR) (Fig. 1h, i, Supplementary Figure S1a). At the steady state, GIR in HFD-fed mice is 12.17 ± 1.09 mg kg^−1^ min^−1^, which is much lower than that in RD-fed mice (22.10 ± 1.23 mg kg^−1^ min^−1^), indicating that insulin sensitivity is decreased in HFD-fed mice (Fig. 1i).

In our experiments, levels of glucose and [6,6-^2^H]glucose were simultaneously measured using mass spectrometer. To distinguish [6,6-^2^H]glucose from natural isotopomers of glucose (M + 2), we used a high resolution mass spectrometer (Orbitrap) coupled with an ultra-high performance liquid chromatography (UPLC) system. It was recently reported that the Orbitrap machine has a mass resolution of 100,000, and can therefore resolve isotopic fine structures^13^. We performed this assay based on a mass resolution of 140,000, which enables us to differentiate oxygen-18 (^18^O), ^13^C and ^2^H isotopes of glucose (M + 2), thereby improving the detection sensitivity of exogenous [6,6-^2^H]glucose. As shown in the left panel of Fig. 1j, no natural _2_H_2_ isotope was detected in serum before [6,6-^2^H]glucose infusion. Natural ^18^O and ^13^C_2_ isotopic peaks were clearly resolved with mass errors of less than 1 ppm. However, when we analyzed a standard mixture solution containing 1 μg ml^−1^ [6,6-^2^H]glucose and 1 mg ml^−1^ glucose (1:1000), a signal from [6,6-^2^H]glucose with 0.41 ppm mass error appeared which was separate from the ^18^O and ^13^C_2_ isotopic peaks (right panel of Fig. 1j).

Next, we measured the relative levels of [6,6-^2^H]glucose and glucose from mice fed with a RD or HFD (Fig. 1k). [6,6-^2^H]glucose and glucose were quantified respectively using the calibration curves in Supplementary Table S1. It turned out that the ratio of [6,6-^2^H]glucose and glucose can be represented using the ratio of the corresponding chromatographic areas. As expected, the [6,6-^2^H]glucose levels were more dramatically decreased in mice during the insulin clamp than in the basal state (Fig. 1k). In addition, plasma enrichment, expressed as atom percent excess (APE), was much higher in the basal state and lower in the insulin clamp state in RD-fed mice than in HFD-fed mice (Fig. 1l). In the basal state, there is lower hepatic glucose production in RD-fed mice than in HFD-fed mice, and this is much clearer during the insulin clamp, indicating a failure of insulin suppression on HGP in HFD-fed mice as a reflection of hepatic insulin resistance (Fig. 1m, n). The decreased glucose disposal rate (GDR) in HFD-fed mice indicated systemic insulin resistance, while insulin-stimulated GDR (IS-GDR) and insulin-induced suppression of plasma FFA levels reflected the relative insulin resistance in muscle and white adipose tissue (WAT) of HFD-fed mice, respectively (Fig. 1o, p, Supplementary Figure S1b). The reduced insulin sensitivity was further confirmed by immunoblots of pAKT in liver, skeletal muscle and WAT of mice fed a HFD (Supplementary Figure S1c). Altogether, these data clearly demonstrated that our method, using [6,6-^2^H]glucose as a tracer, successfully evaluates the impaired insulin action and glucose metabolism in diet-induced obese mice.

In summary, we have successfully developed a sensitive hyperinsulinemic-euglycemic clamp method using [6,6-^2^H]glucose as a tracer followed by identification with a high resolution mass spectrometer, which generates similar results to methods based on the radioactive tracer^9, 10, 14^. The radioactive tracer is harmful to the environment, very expensive, and restricted by regulations. Previously reported methods in humans used a high dosage of [6,6-^2^H]glucose infusion, which had a non-negligible effect on glucose metabolism^4–8^. In contrast, we used a very low dose of [6,6-^2^H]glucose, which had no effect on blood glucose levels, and was distinguished from the natural isotopomers of glucose by high resolution mass spectrometry. Therefore, this method makes the low dosage infusion of stable isotope-labeled glucose in humans possible for further sensitive detection and is suitable for use in medical research. Our method can also be combined with serum metabolomics^15^ based on high-throughput analyses by mass spectrometry, which would allow the analysis of many metabolites in parallel.

Methods are available in Supplementary Information.

## Electronic supplementary material

Evaluation of Insulin Sensitivity by Hyperinsulinemic-Euglycemic Clamps Using Stable Isotope-Labeled Glucose
