# Supplementary material for: Evaluation of insulin sensitivity by hyperinsulinemic-euglycemic clamps using stable isotope-labeled glucose
Source: Cell Discov. 2018 Apr 17;4:17. doi: 10.1038/s41421-018-0016-3 (PMC5902507; doi:10.1038/s41421-018-0016-3)
Supplement: Supplementary file 1 — Evaluation of Insulin Sensitivity by Hyperinsulinemic-Euglycemic Clamps Using Stable Isotope-Labeled Glucose [file 41421_2018_16_MOESM1_ESM.pdf]

SUPPLEMENTARY TABLE and FIGURE LEGENDS

Table S1. Limit of detection (LOD), limit of quantitation (LOQ) and linearity of glucose and [6,6-^{2}H]-glucose used for analysis of serum samples in this experiment.

Y indicates the peak area of the compound; X indicates the concentration (μg ml^{-1}).

Table S2. Accuracy, intra-day precision and inter-day precision of glucose and [6,6-^{2}H]-glucose at various concentrations.

RSD, relative standard deviation.

Figure S1. Evaluation of insulin sensitivity in mice fed a RD or HFD.

a Plasma insulin levels under basal and clamp conditions are shown.

b Free fatty acid (FFA) suppression is shown.

c Immunoblots showing the reduced pAKT levels in liver, skeletal muscle and white adipose tissue (WAT) extracts of mice fed a HFD.

Data are shown as mean ± s.e.m. ***P* < 0.01, n = 5.

Table S1

Compound	LOD	LOQ	Regression line	R^2	Linear range
[6,6-^{2}H]glucose	10 pg	20 pg	Y = 78635 + 1622410X	0.9937	100 pg - 20 ng
Glucose	40 pg	100 pg	Y = 56285200 + 1978200X	0.9946	100 ng - 4 μg

Table S2

Compound	Concentration (μg ml^{-1})	Intra-day precision (n=3) RSD (%)	Inter-day precision (n=3) RSD (%)	Accuracy (n=3) RSD (%)
[6,6-^{2}H]glucose	0.25	3.5	2.7	9.9
	2.5	3.6	9.4	2.9
	25	2.8	5.0	0.3
Glucose	2.5	4.0	3.8	6.8
	25	1.7	3.5	1.2
	250	1.1	9.5	8.3

Figure S1

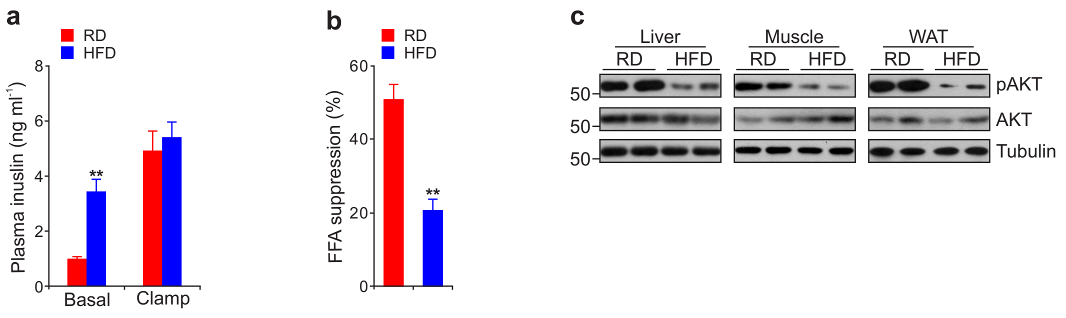

METHODS

Mice

C57BL/6J mice were housed in colony cages under 12-hr light/dark cycle. Mice with diet-induced obesity were obtained by feeding a diet containing 60 kcal% fat (Research Diets, D12492) for 16 weeks. All animal experiments were carried out with the approval of the Animal Care and Use Committee at Tsinghua University.

Hyperinsulinemic-Euglycemic Clamp

Mice were anesthetized with Pentobarbital sodium salt (Sigma, P3761). Hairs at the incision site were removed and skin was disinfected by a betadine scrub. An incision was made in the skin and the right jugular vein was identified. A catheter (PE-10) filled with heparinized saline (200 units heparin ml^{-1} saline) was inserted into the vein toward the side of the chest. A suture was placed at each end of the vessel. The hairs were removed from the area around the shoulder blades and the catheter was tunneled under the skin from the right jugular to the interscapular incision on the back. The catheter was plugged with a stainless steel plug and settled on the back. All skin wounds were sutured and the mouse was allowed to recover for 4-5 days. Mice that lost < 4% of their precannulation weight after recovery were used for clamp studies.

The mice were fasted overnight, then weighed to calculate the insulin dose and placed in a restrainer. The setup and time-line for the experiment are shown in Figure 1F. An equilibration syringe was connected to the catheter and an initial

bolus of [6,6-^{2}H]glucose (600 μg kg^{-1}) was administered, followed by continuous infusion of [6,6-^{2}H]glucose (30 μg kg^{-1} min^{-1}). Isotopic enrichment was achieved at approximately 60 min after the onset of isotope infusion, and blood samples for isotope measurements were collected at t = -10 and 0 min. Following this basal infusion period, insulin was constantly infused at the rate of 6 mU kg^{-1} min^{-1} until termination of the study. [6,6-^{2}H]glucose was infused together with glucose at various rates until the blood glucose concentration reached a constant level of about 100 ± 5 mg dl^{-1}. Blood samples were taken at t = 110 and 120 min.

Mass spectrometry

All plasma samples (15 μl) were deproteinized by gently mixing them with 60 μl cold methanol (pre-chilled at -80 ºC) and incubating for 1-2 hrs at - 80 ºC. The samples were then centrifuged with 14,000 g at 4 ºC for 10 min. The supernatant was transferred to a new tube and lyophilized to produce a pellet. High resolution mass spectrometry (Q Exactive, Thermo) coupled with Ultimate 3000 ultra-high performance liquid chromatography (UPLC) was used for the analysis. Negative ion mode with a mass resolution of 140,000 was performed for glucose detection. Flow rate of sheath gas and aux gas was set as 35 and 10 respectively. Spray voltage was 2.8 kV. For LC separation, an amide column (2.1 mm × 100 mm, Waters) was used for analysis of glucose. Column temperature was 40 ºC. Mobile phases contained 80% acetonitrile in A and 30% acetonitrile in B with 5 mM ammonium acetate as modifier. The flow rate was

250 μL min^{-1} and the gradient was as follows: 0-3 min: 10% B; 3-4 min: 10-60% B; 4-6 min: 60% B; 6.1-8 min: 10% B. A total of 2 μL sample was injected for analysis. Accurate masses of 179.0561 and 181.0686 with mass tolerance of 3 ppm were used for extraction of glucose and [6,6-^{2}H]glucose in negative mode. For the calibration curve, concentrations of 5, 10, 25, 50, 100, 250, 500, 1000, 2500, 5000, 10000, 25000 ng ml^{-1} for [6,6-^{2}H]glucose and 1, 2, 5, 10, 20, 50, 100, 200, 500, 1000, 2500, 5000 μg ml^{-1} for glucose were prepared in 80% methanol. The precision, accuracy, sensitivity and linearity of this method were evaluated are shown in Supplementary Table S1 and Table S2.

Calculation

Under steady-state conditions, the rate of glucose appearance is equal to the rate of disappearance. Our calculations are all based on Steele's equations for steady-state conditions[16, 17]. Because [6,6-^{2}H]glucose is not massless, the glucose disposal rate (GDR) is equal to the constant isotope infusion rate (F) divided by the isotope enrichment (atom percent excess, APE) minus F[16, 17]. During the basal infusion period, while there is no glucose infusion, GDR is equal to the rate of hepatic glucose production (HGP). During infusion of unlabeled glucose mixed with [6,6-^{2}H]glucose, GDR is equal to the sum of the rate of HGP and the glucose infusion rate (GIR). Therefore, the HGP is calculated by subtracting the GIR from the calculated GDR. The calculation was performed as indicated in Figure 1G.

Immunoblotting

The assays were performed as previously described [9, 18]. In brief, mouse tissues were homogenized in RIPA buffer (25 mM Tris-HCl pH 7.4, 1% NP-40, 1% sodium deoxycholate, 0.1% SDS, 150 mM NaCl). Protein concentrations were determined using the BCA Protein Assay Kit (23227, Thermo Fisher). Samples were loaded on SDS-PAGE gels and then transferred to nitrocellulose membranes. Immunoblotting was done in gelatin buffer (50 mM Tris HCl pH 7.4, 150 mM NaCl, 5 mM EDTA, 0.05% Tween-20) with the corresponding antibodies. The rabbit polyclonal antibody against anti-pAKT (13038) was purchased from Cell Signaling Technology. The rabbit polyclonal antibody against anti-AKT (SC-8312) was from Santa Cruz. The mouse monoclonal antibody against anti-Tubulin (T6199) was from Sigma-Aldrich.

Measurement of Plasma insulin and free fatty acids

Plasma insulin (Mercodia, 10-1247-01) and free fatty acid levels (Wako, 294-636) were measured according to the manufacturer's instructions.

Statistical analyses

Age- and weight-matched male mice were randomly assigned for the experiments. The number of animals used in each experiment is outlined in the corresponding figure legends. No animals were excluded from statistical analyses, and the investigators were not blinded in the studies. All studies were performed on at least three independent occasions. Results are reported as mean ± s.e.m. Comparison of different groups was carried out using two-tailed unpaired Student's *t*-test. Differences were considered statistically significant at

$P < 0.05$.
